# Giant Nonequilibrium Valley Control via Spin‐Selective Hot‐Carrier Transfer in an Antiferromagnetic van der Waals Stack

**DOI:** 10.1002/adma.75103

**Published:** 2026-09-29

**Authors:** Ke Xiao, Jiabao Yang, Kai Feng, Yicheng Guan, James Caleb Peters, Ramona Hoffmann, Niels Schröter, Stuart S. P. Parkin

**Affiliations:** ^1^ NISE Department Max Planck Institute of Microstructure Physics Halle (Saale) Germany; ^2^ Physics Department and State Ley Laboratory of Quantum Optical Materials The University of Hong Kong Hong Kong China; ^3^ Martin‐Luther‐Universität Halle‐Wittenberg Halle (Saale) Germany

**Keywords:** antiferromagnetism, circular polarization, exciton, heterojunction, magnet, materials science, semiconductor, spectroscopy, spin polarization

## Abstract

Spin‐selective hot‐carrier transfer at interfaces between two‐dimensional (2D) semiconductors and magnets offers a nonequilibrium route to manipulate spin and valley degrees of freedom. In van der Waals heterostructures, such transfer can be enabled by type‐III band alignment together with magnetic‐field‐induced spin polarization of the bands in adjacent magnetic layers. Yet, continuous and robust valley control with high magnetic‐field susceptibility remains challenging, particularly beyond binary switching in out‐of‐plane easy‐axis magnets. Here we fabricate a CrSBr/WSe_2_/CrSBr heterostructure, where two semiconductor‐magnet interfaces promote efficient hot‐carrier transfer. Utilizing valley‐resolved magneto‐photoluminescence spectroscopy, we reveal a pronounced field‐sign‐asymmetric response and a magnetic‐state‐dependent evolution of emergent spectral features that track the spin configuration of multilayer CrSBr via spin‐selective hot‐carrier transfer. This mechanism enables giant, continuous tuning, and magnetic‐state‐dependent reversal of the degree of circular polarization (DoCP), reaching ∼ 40% for excitons and ∼ 80% for trion emission. Our results establish spin‐polarized interlayer hot‐carrier transfer as an efficient knob for engineering valley polarization and coherence in TMDs, and highlight antiferromagnet‐based stacks as a versatile platform for magnetically programmable quantum optoelectronic functionalities.

## Graphical abstract

Spin‐selective hot‐carrier transfer across CrSBr/WSe_2_/CrSBr interfaces couples the continuously tunable magnetic configuration of antiferromagnetic CrSBr to the valley degrees of freedom of monolayer WSe_2_, enabling giant nonequilibrium valley control and magnetic‐state‐dependent reversal of circularly polarized emission. This work establishes antiferromagnet‐based van der Waals stacks as a versatile platform for magnetically programmable valleytronic and quantum optoelectronic functionalities.

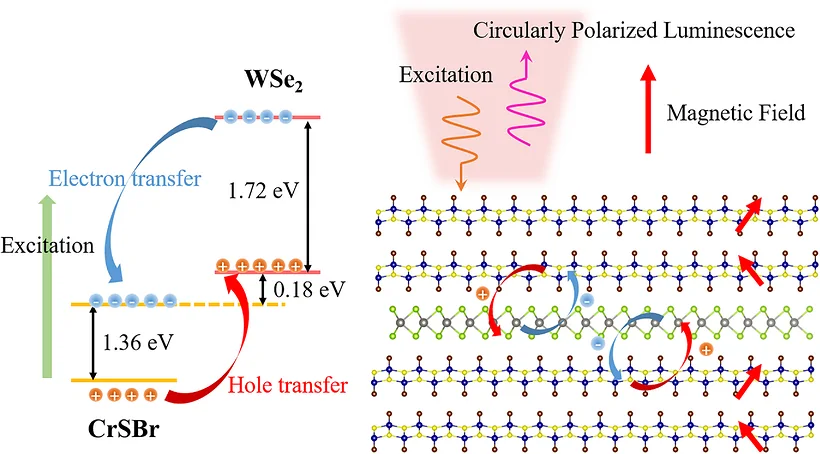

## 1 Introduction

Monolayer transition‐metal dichalcogenides (TMDs) lack inversion symmetry and exhibit strong spin–orbit coupling, giving rise to two energy‐degenerate yet inequivalent valleys at *K* and *‐K* at the corners of the Brillouin zone [1, 2]. This valley degree of freedom provides an additional handle for quantum‐state manipulation beyond charge and spin. Selectively pumping one of the valleys with circularly polarized light will induce imbalanced carrier distribution between the *K* and *‐K* valleys [3, 4, 5]. In pristine TMDs however, valley polarization is limited by inevitable intervalley scattering and depolarization processes [6, 7, 8, 9, 10, 11]. Although near‐resonant excitation [12, 13] and strain engineering [14, 15, 16] can enhance valley polarization, the former relies on stringent excitation conditions, while the latter often suffers from limited reproducibility and scalability, hindering practical valleytronic implementations. Beyond high polarization, continuous, and broadly tunable control of the emitted polarization is also desirable for valley‐based information encoding.

A promising strategy is to couple TMDs to van der Waals (vdW) magnets, where interfacial exchange and spin‐dependent carrier transfer can imprint magnetic selectivity onto valley populations. Spin‐selective charge transfer has been demonstrated in heterostructures based on CrI_3_ [17, 18, 19, 20], CrBr_3_ [21–23], Fe_3_GeTe_2_ [24, 25], and Fe_3_GaTe_2_ [26, 27]. Yet these magnets typically possess an out‐of‐plane easy axis, so sweeping the magnetic field mainly toggles between two discrete spin configurations (up or down), leading to essentially binary transfer pathways. Recently, CrSBr has emerged as an air‐stable layered vdW antiferromagnet with Néel temperatures of ∼150 K (bilayer) and ∼132 K (bulk) [28, 29]. CrSBr hosts in‐plane ferromagnetic order within each layer with an easy axis along the crystallographic *b* direction, together with antiferromagnetic interlayer coupling. Several studies have reported charge transfer between monolayer TMDs and CrSBr [30, 31, 32, 33, 34], often emphasizing defect‐related or localized excitonic states. However, how the valley polarization of intrinsic excitons in WSe_2_ can be tuned by an external magnetic field and in particular the origin of field‐sign‐asymmetric photoluminescence (PL) responses, remains largely unexplored.

Here we address these questions using a CrSBr/WSe_2_/CrSBr heterostructure, where two semiconductor‐magnet interfaces enable highly efficient interlayer hot‐carrier transfer. Here, “hot‐carriers” refer to photoexcited carriers that undergo interfacial transfer before carrier‐lattice equilibration [35]. Using valley‐resolved magneto‐PL spectroscopy, we show that interfacial transfer suppresses valley coherence, leading to a reduced degree of linear polarization (DoLP). Under circularly polarized excitation, we observe a pronounced field‐sign asymmetry and giant continuous tunability of valley polarization or degree of circular polarization (DoCP), which we attribute to spin‐selective hot‐carrier transfer governed by the spin configuration of multilayer CrSBr. These results establish antiferromagnet‐based van der Waals stacks as a versatile platform for magnetically programmable control of valley degrees of freedom and quantum optoelectronic functionalities.

## 2 Results

### 2.1 Raman Fingerprints of CrSBr and WSe_2_

Figure 1a schematically illustrates the multilayer CrSBr/monolayer (1L) WSe_2_/multilayer CrSBr heterostructure investigated in this work, and Figure 1b shows a representative optical micrograph of a fabricated device. Individual flakes were mechanically exfoliated and assembled inside a nitrogen‐filled glovebox using a dry‐transfer technique (Methods). The 1L‐WSe_2_ is sandwiched between two multilayer CrSBr flakes, with the top and bottom CrSBr aligned to the same crystallographic orientation. The thicknesses of the top and bottom CrSBr flakes were determined to be 10.6 and 8.1 nm, corresponding to approximately 13 and 10 layers, respectively (Figure S2a–c). Both flakes are therefore in the thick, bulk‐like A‐type antiferromagnetic regime. Cross‐sectional high‐angle annular dark‐field scanning transmission electron microscopy (HAADF‐STEM) further confirms that monolayer WSe_2_ is embedded between the two CrSBr flakes with sharp interfaces and well‐defined van der Waals gaps (Figure S2d‐f). The crystallographic alignment of the two CrSBr flakes is independently verified by polarization‐resolved Raman spectroscopy (Figure S3) and supported by cross‐sectional HAADF‐STEM measurements. The heterostructures were fabricated on SiO_2_/Si substrates and measured in a temperature‐controlled, magnetic‐field‐variable cryostat (Methods). Unless otherwise noted, optical measurements were performed at 2 K in a home‐built confocal microscope under 633 nm continuous‐wave He‐Ne laser excitation, with an estimated spot radius of ∼2 µm.

**FIGURE 1:**
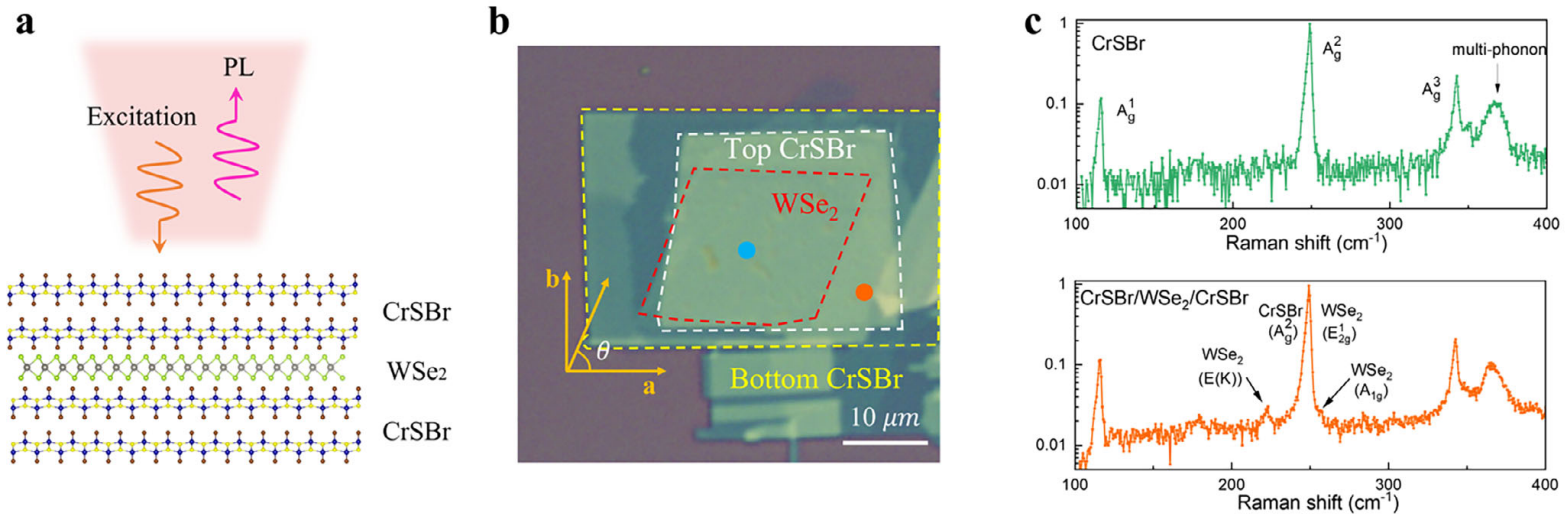
Device architecture and Raman fingerprints of the CrSBr/1L‐WSe_2_/CrSBr heterostructure. (a) Schematic illustration and (b) optical image of the multilayer CrSBr/1L‐WSe_2_/multilayer CrSBr heterostructure. The bottom CrSBr flake, 1L‐WSe_2,_ and top CrSBr flake are outlined by yellow, red, and white dashed lines, respectively. The top and bottom CrSBr flakes were aligned to the same crystallographic orientation. Gold axes indicate the crystallographic a and b directions of CrSBr. Scale bar: 10 µ*m* (c) Raman spectra acquired on bare CrSBr (top; orange spot in (b)) and on multilayer CrSBr/1L‐WSe_2_/multilayer CrSBr heterostructure (bottom; blue spot in b).

Figure 1c compares the Raman spectra acquired from a bare CrSBr region (top panel) and the heterostructure (bottom panel), as indicated by orange and blue circles in Figure 1b. Three prominent peaks at 121 ($A_{g}^{1}$), 250 ($A_{g}^{2}$), and 346 cm^−1^ ($A_{g}^{3}$) are observed, the broad peak at around 375 cm^−1^ is attributed to a multi‐phonon process, consistent with prior reports [36, 37, 38]. The characteristic phonon modes of monolayer WSe_2_ (*E*′ ≈ 249 cm^−1^, $A_{1}^{\prime }\approx$ 251 cm^−1^) overlap spectrally with the CrSBr $A_{g}^{2}$ mode and are weaker in intensity, hindering a clear separation. Nevertheless, a weak shoulder on the high‐frequency side of $A_{g}^{2}$ can be resolved and is assigned to the WSe_2_ $A_{1}^{\prime }$ mode. In addition, a second‐order Raman feature around 225 cm^−1^ is observed and attributed to the two‐phonon process (*E*(*K*)) involving phonons from the ± K valleys [39].

### 2.2 Anisotropy and Loss of Valley Coherence

We performed linear polarization‐resolved PL measurements (Methods), where the excitation and detection polarizations were rotated together in either co‐polarized (XX) or cross‐polarized (XY) configurations (schematic in Figure S1a). Figure 2a,b show the polarization‐angle‐dependent PL intensity maps for XX and XY detection, respectively, with the excitation polarization angle (θ) referenced to the CrSBr *a*‐axis (Figure 1b). The dashed lines in Figure 2a,b mark excitation polarized along the CrSBr *a*‐ (yellow) and *b*‐axis (brown). Two emission bands dominate the spectra: A lower‐energy feature at ∼ 1.36 eV together with a broader low‐energy peak from CrSBr exciton, and a pronounced peak at ∼1.71 eV assigned to WSe_2_ emission, comprising the neutral exciton and a trion or more accurately a Fermi‐polaron [40, 41, 42] contribution.

**FIGURE 2:**
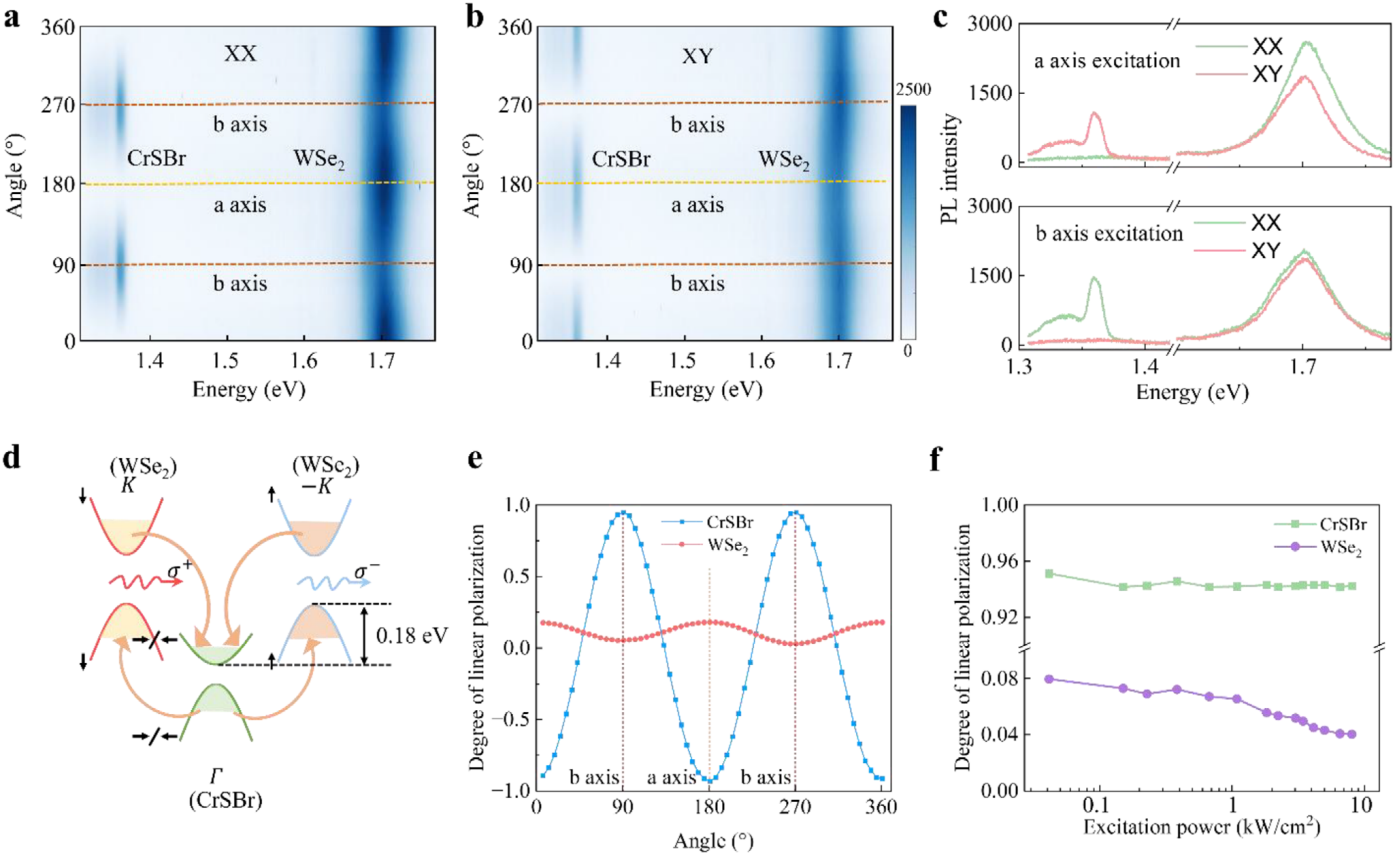
Hot‐carrier‐transfer‐induced suppression of valley coherence at zero magnetic field. (a, b) Polarization‐angle‐dependent PL intensity maps measured in co‐polarized (XX) (a) and cross‐polarized (XY) (b) configurations. The excitation polarization angle (θ) is defined relative to the CrSBr a‐axis (Figure 1b). Panels a and b share the same color scale, shown at the right of panel b. Yellow and brown dashed lines mark excitation along a and b axes, respectively. The emission near 1.36 eV originates from CrSBr, whereas the feature near 1.71 eV originates from WSe_2_. (c) Representative PL spectra in XX (light green) and XY (light red) for excitation along the CrSBr a‐axis (top) and b‐axis (bottom). (d) Schematic of type‐III band alignment with broken gap 0.18 eV and interfacial hot‐carrier transfer at *B_z_* = 0, where photoexcited electrons transfer from WSe_2_ to CrSBr, while photoexcited holes transfer from CrSBr to WSe_2_, providing an additional valley‐dephasing channel for WSe_2_. Linearly polarized emission from WSe_2_ arises from a coherent superposition of photons emitted by *K* and *‐K* valley excitons with a well‐defined relative phase. Black arrows denote the spin orientation of the relevant bands. (e) Excitation‐polarization‐angle dependence of the DoLP for WSe_2_ (light red) and CrSBr (blue), showing a sinusoidal modulation with maxima along the a and b axes, respectively. (f) Excitation‐power dependence of the DoLP under b‐axis excitation. The DoLP of CrSBr (light green) remains nearly unchanged over the measured range, whereas the DoLP of WSe_2_ (purple) decreases monotonically with increasing power.

Both XX and XY maps exhibit a clear two‐fold angular modulation (polar plots of integrated PL intensity are shown in Figure S2a), consistent with the in‐plane anisotropy of CrSBr, whose crystal structure belongs to point group *D*_2*h*_. We quantify the modulation by the anisotropy ratio $AR=\frac{I_{max}}{I_{min\;}}$. CrSBr shows strong anisotropy, with $AR_{CrSBr}^{XX}=39.7$, $AR_{CrSBr}^{XY}=28.0$, whereas, WSe_2_ shows weak anisotropy $AR_{WSe_{2}}^{XX}=1.2$, $AR_{WSe_{2}}^{XY}=1.05$, in contrast with a bare monolayer WSe_2_ sample where no anisotropy is observed [43]. Notably, the major anisotropy axes of CrSBr and WSe_2_ are opposite in both XX (CrSBr: *b*‐axis, WSe_2_: *a*‐axis) and XY (CrSBr: *a*‐axis, WSe_2_: *b*‐axis) configurations (Figure S4). This behavior indicates that weak WSe_2_ anisotropy originates from interlayer coupling in the heterostructure rather than intrinsic lattice symmetry.

Representative spectra for excitation polarized along the CrSBr *a*‐axis (top) and *b*‐axis (bottom) are shown in Figure 2c. For CrSBr, excitation along the *a*‐axis yields a stronger signal in the cross‐polarized channel XY(*ab*), while the co‐polarized XX(*aa*) channel is strongly suppressed;the trend reverses for excitation along the b‐axis, where XX(*bb*) is dominant and XY(*ba*) becomes negligible. This behavior indicates that CrSBr excitons predominantly couple to an optical dipole aligned along the *b*‐axis, no matter which direction the excitation polarization is. In contrast, WSe_2_ exhibits a weaker, but consistent polarization dependence: XX always exceeds XY, whereas the XX‐XY contrast is minimized when exciting along the *b*‐axis of CrSBr. This trend rules out a purely photonic origin (e.g., linear dichroism filtering by CrSBr) and instead points to an excitation‐axis‐dependent interfacial process.

We further quantify polarization retention by the degree of linear polarization, $DoLP=\frac{I^{XX}-I^{XY}}{I^{XX}+I^{XY}}$, where *I^XX^* and *I^XY^* denote the emission intensities in XX and XY channels, respectively. In monolayer TMDs, DoLP is commonly used as a proxy for valley coherence [44, 45] (Figure 2d), where left‐ (σ^+^) and right (σ^−^) circularly polarized light from *K* and *‐K* valley excitons form a coherent superposition with a well‐defined relative phase, such that linearly polarized excitation yields linearly polarized emission. In CrSBr/1L‐WSe_2_/CrSBr heterostructures, the type‐III band alignment, where the CrSBr conduction‐band minimum lies approximately 0.18 eV below the WSe_2_ valence‐band maximum [46] [More details about band‐edge energies of WSe_2_ and CrSBr are shown in Figure S5 and Table S1], enables efficient hot‐carrier transfer with electrons transferring from WSe_2_ to CrSBr, and holes transferring from CrSBr to WSe_2_. Such interlayer transfer provides an additional valley‐dephasing pathway and suppresses the DoLP of WSe_2_. Moreover, consistent with the *b*‐axis‐oriented excitonic dipole discussed above, *b*‐axis excitation generates a larger number of photoexcited carriers in CrSBr and enhances interfacial transfer, yielding the minimum WSe_2_ DoLP near *b*‐axis excitation and producing a sinusoidal DoLP modulation with polarization angle as shown in Figure 2e. To directly test the role of hot‐carrier transfer, we fixed the excitation polarization along the *b*‐axis and performed excitation‐power‐dependent measurements (Figure S6). The DoLP of CrSBr remains nearly unchanged at high levels from 0.04 to 8 kW/cm^−2^, whereas the DoLP of WSe_2_ excitons decrease monotonically with increasing power, consistent with progressively stronger interfacial transfer and enhanced valley dephasing. In contrast, the WSe_2_ trion feature exhibits a much lower DoLP across the same power range (Figure S6c), consistent with the orthogonality of the relevant hole states in the *K* and *‐K* valleys reported previously [44].

### 2.3 Ultrafast Interfacial Hot‐Carrier Transfer

To directly resolve the interfacial carrier‐transfer dynamics, we performed low‐temperature femtosecond transient‐reflection measurements on the CrSBr/WSe_2_/CrSBr heterostructure (Figure 3). A 1.46 eV pump was used to selectively excite CrSBr, as its photon energy lies above the CrSBr optical gap (∼1.36 eV), but below the WSe_2_ A‐exciton energy (∼1.72 eV) (Figure 3a). Accordingly, bare WSe_2_ shows no transient feature near the A‐exciton resonance (Figure 3b). In contrast, a pronounced transient response emerges at the WSe_2_ A‐exciton energy in the CrSBr/WSe_2_/CrSBr heterostructure (Figure 3b–d), directly demonstrating interfacial carrier transfer from CrSBr to WSe_2_ under the type‐III band alignment.

**FIGURE 3:**
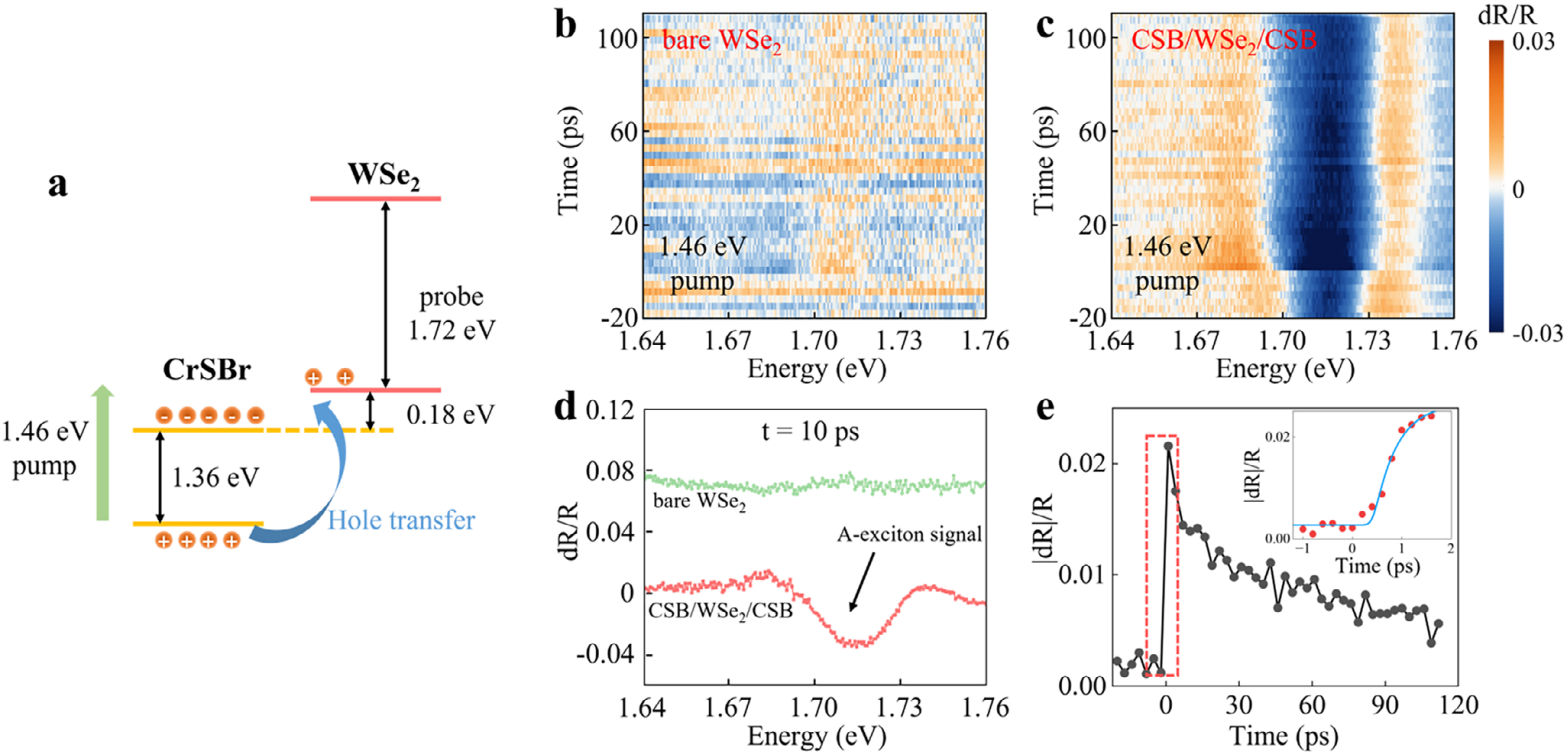
Ultrafast interfacial charge transfer in the CrSBr/WSe_2_/CrSBr heterostructure. (a) Energy‐level scheme. A 1.46 eV pump (green) lies above the CrSBr optical gap (∼1.36 eV), but below the WSe_2_ gap (∼1.72 eV), selectively photoexciting CrSBr; under the type‐III (broken‐gap) alignment, photoexcited holes transfer from CrSBr into the WSe_2_ valence band (blue arrow), and are probed at the WSe_2_ A‐exciton energy (∼1.72 eV). (b,c) Transient‐reflection (dR/R) maps versus probe energy and pump‐probe delay under 1.46 eV excitation for (b) bare WSe_2_ and (c) the CrSBr/WSe_2_/CrSBr heterostructure. No clear feature appears near the A‐exciton for bare WSe_2_, whereas a clear A‐exciton bleach develops in the heterostructure. (d) dR/R spectra at *t* = 10 ps for bare WSe_2_ (green) and the heterostructure (red). (e) Time trace of the A‐exciton signal magnitude |dR|/R. Fitting the rising edge (dashed box, measured with fine time steps in the inset) with an exponential convolved with the ∼120 fs instrument response yields a transfer time constant of τ ≈ 0.46 ± 0.17 ps (sub‐picosecond).

The WSe_2_ A‐exciton transient develops within the first picosecond after excitation (Figure 3e). Fitting the rising dynamics with an exponential function convolved with the ∼120 fs instrument response yields a transfer time constant of τ = 0.46 ± 0.17 ps, comparable to the sub‐picosecond hot‐hole transfer reported at the CsPbI_3_/WSe_2_ interface [47]. This sub‐picosecond timescale demonstrates that interfacial transfer occurs in the early nonequilibrium regime, before carrier‐lattice equilibration (Figure S7).

To further examine the competition between interfacial transfer and WSe_2_ exciton formation, we performed additional transient‐reflection measurements using a 2.91 eV (426 nm) pump, which directly excites both WSe_2_ and CrSBr (Figure S8). Under identical excitation conditions, the WSe_2_ A‐exciton transient in the heterostructure is strongly suppressed to approximately one third of that in bare WSe_2_, consistent with an additional ultrafast interfacial transfer channel that competes with the formation and population of WSe_2_ excitons. Time‐resolved PL measurements further reveal an instrument‐limited fast decay component of τ_1_  <  20 ps and a slower decay component of τ_2_  =  303  ±  16 ps (Figure S9), which is substantially longer than the ∼0.46 ps interfacial transfer time. Together, these measurements establish the ultrafast, nonequilibrium nature of the interfacial carrier transfer and its competition with the early‐stage exciton dynamics in WSe_2_.

### 2.4 Field‐Sign Asymmetry Linked to the CrSBr Magnetic States

We next examine how the interfacial hot‐carrier transfer correlates with the magnetic state of CrSBr. Figure 4 summarizes circular‐polarization‐resolved PL spectra of the heterostructure under an out‐of‐plane magnetic field *B_z_*. Measurements were performed with co‐aligned excitation and detection polarizations (Methods and Figure S1b), and all four circular combinations (σ^+^σ^+^, σ^+^σ^−^, σ^−^σ^+^,σ^−^σ^−^) are shown in Figure 4a–d, respectively. The detected circular polarization is verified to be insensitive to Faraday rotation from the cryostat window (Figure S10).

**FIGURE 4:**
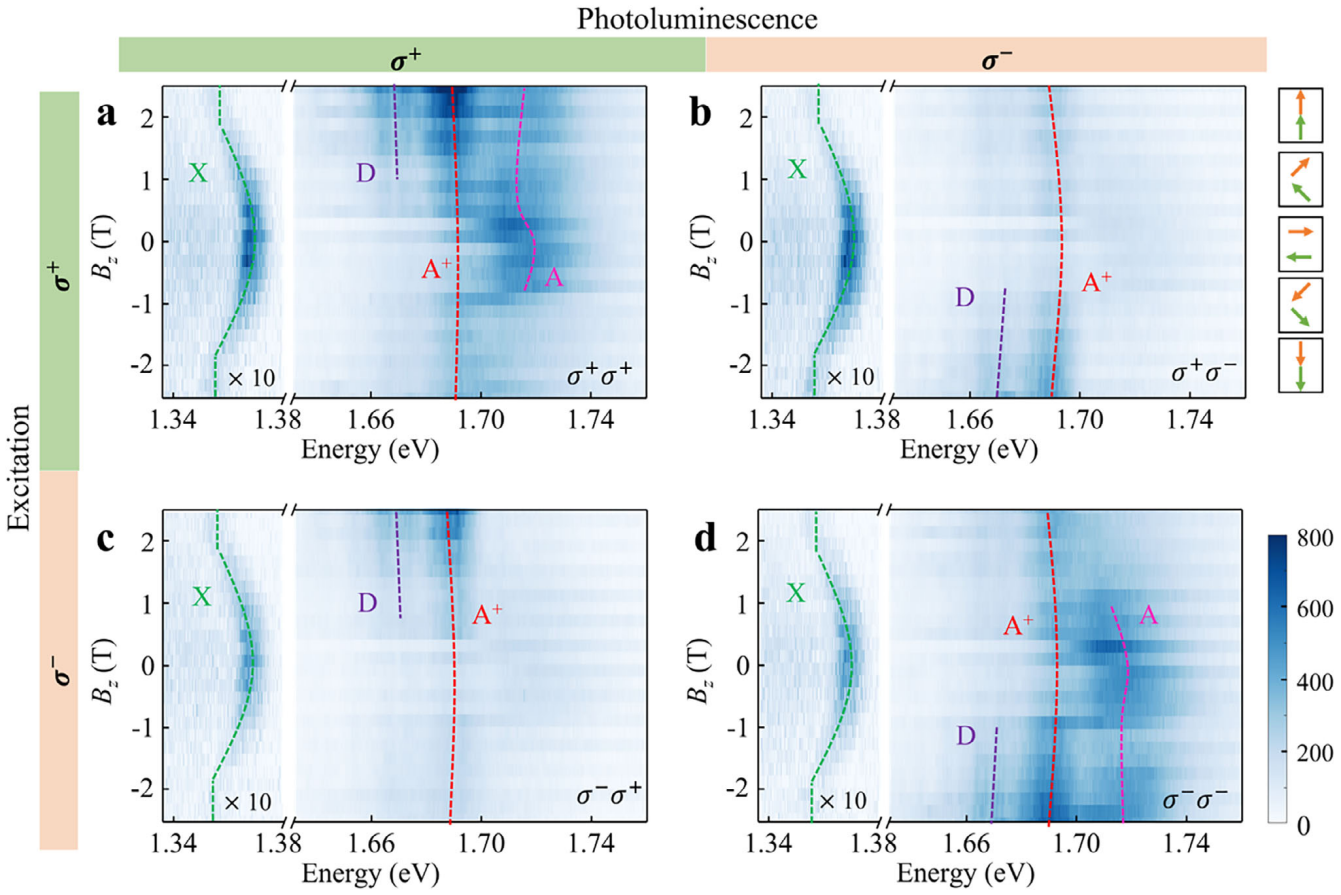
Magneto–valley‐resolved PL of the CrSBr/1L‐WSe_2_/CrSBr heterostructure. (a–d) Magnetic‐field‐dependent PL intensity maps measured under the four excitation/detection helicity combinations: σ^+^σ^+^ (a), σ^+^σ^−^ (b), σ^−^σ^+^ (c), and σ^−^σ^−^ (d). The CrSBr intensity is scaled by ×10 for comparison with WSe_2_. All panels share the same color scale (shown to the right of panel (d)). The CrSBr emission (labelled *X*, left spectral window) exhibits an approximately quadratic energy shift with out‐of‐plane magnetic field *B_z_* (green dashed guide). The CrSBr peak energy saturates above |*B_z_* | ≈ 1.8 T, consistent with a field‐aligned spin configuration (schematic at the right panel (b)). Purple, red, and pink dashed lines are rough guides to the eye highlighting the field evolution of WSe_2_ dark exciton (labelled as *D*), trion feature (labelled as *A^+^*), and bright A‐exciton (labelled as *A*).

We first focus on the CrSBr emission. In the (σ^+^σ^+^) map (Figure 4a), the CrSBr exciton peak (labelled *X*) exhibits a continuous redshift as |*B_z_* | increases from 0 to ∼ 1.8 T (CrSBr intensity scaled by 10 for comparison with WSe_2_). The quadratic‐like field dependence of the CrSBr PL energy reflects the evolution of the CrSBr spin configuration under hard‐axis field and agrees with previous reports [28, 48]. This quadratic‐like dependence is attributed to the enhanced interlayer electronic hopping by gradual overlap of spin canting/reorientation between neighboring layers gradual and the band splitting by external magnetic field [49]. Above ∼1.8 T, the peak energy remains unchanged (green dashed guide in Figure 4a), consistent with a field‐driven approach to a fully aligned spin state (schematic in the right panel of Figure 4b). The same peak‐position evolution is observed in all four polarization channels (green dashed guides in Figure 4a–d), making the CrSBr PL peak energy an experimental marker of the CrSBr magnetic configuration in the measured field range. Notably, the CrSBr exciton energy exhibits the same field evolution for all excitation/detection helicity combinations, indicating that the CrSBr magnetic configuration is not measurably modified by the excitation helicity. This argues against a helicity‐induced change of the CrSBr magnetic state as the origin of the asymmetric WSe_2_ response [50]. It should also be noted that the CrSBr layer number can influence this magneto‐optical response primarily through the magnetic‐field scale of the spin reorientation. At least two layers are required to establish the A‐type antiferromagnetic interlayer coupling, while thinner CrSBr, such as bilayers exhibits a somewhat smaller saturation field than thick, bulk‐like flakes [28]. In our device, both CrSBr flakes are thick multilayers (∼13 and ∼10 layers), and their bulk‐like spin alignment near ∼1.8 T coincides with the characteristic evolution of the WSe_2_ DoCP discussed below.

In the WSe_2_ spectral window, three features near 1.715, 1.695, 1.662 eV are resolved and assigned to the bright A exciton (labelled as *A*), the positively charged trion (labelled as *A^+^*), and the spin‐forbidden dark exciton (labelled as *D*), respectively, by comparison with valley‐resolved PL results for the *h*BN/WSe_2_/*h*BN sample (Figures S11 and S12) and prior reports [9, 51]. At *B_z_* = 0, the A‐exciton is clearly visible in the co‐polarized channels (σ^+^σ^+^ and σ^−^σ^−^, Figure S11). With increasing |*B_z_* |, *A^+^* becomes increasingly prominent (e.g., Figure 4a,d), indicating a field‐dependent change in the carrier population in WSe_2_, consistent with enhanced interlayer charge transfer across the interface.

Strikingly, the polarization‐resolved WSe_2_ response exhibits a pronounced asymmetry with respect to the sign of B (Figure 4a–d), in stark contrast to the nearly symmetric response of bare monolayer WSe_2_ (Figures S13 and S14). This characteristic field‐sign‐asymmetric response persists at an elevated temperature of 50 K, although with a reduced magnitude compared with 2 K (Figures S15 and S16), further supporting its magnetic origin. These optical and magneto‐optical responses are spatially reproducible across the heterostructure, with comparable PL spectra and DoLP values, as well as consistent field‐sign asymmetry and characteristic DoCP evolution near |*B_z_* | ∼1.8 T, observed at multiple positions (Figures S17 and S18). Combined with the sub‐picosecond interfacial carrier transfer established above, these field‐, helicity‐, and temperature‐dependent responses support a spin‐selective hot‐carrier‐transfer mechanism governed by the magnetic configuration of CrSBr.

Under *B_z_* > 0, σ^+^ excitation predominantly prepares carriers in the *K* valley, with a smaller *‐K* valley population arising from intervalley scattering (Schematic in Figure 5a). In the presence of a spin‐polarized band in CrSBr under external magnetic field [49], interlayer transfer becomes valley and spin dependent, producing a redistribution of carriers between *K* and *‐K* (Figure 5b). Consistent with this picture, *A* remains comparatively robust in σ^+^σ^+^, while the *A^+^* contribution is enhanced, as supported by peak fits shown in the upper panel of Figure 5c. For *B_z_* < 0, σ^+^ excitation produces a similar initial valley‐selective hot‐carrier distribution, but the allowed transfer channels invert, that means only carriers originating from the *K* valley can efficiently transfer to CrSBr, with the reversed spin polarized band in CrSBr (Figure 5d). The resulting steady‐state carrier repopulation differs markedly (Figure 5e), yielding substantial hole occupation in the *K* valley valence band, but reduced electron occupation in the *K* valley conduction band. Consequently, *A* remains present (though weaker) in σ^+^σ^+^, whereas *A^+^* becomes comparatively more pronounced in σ^+^σ^−^, consistent with the availability of additional holes that favor trion formation via intervalley pairing.

**FIGURE 5:**
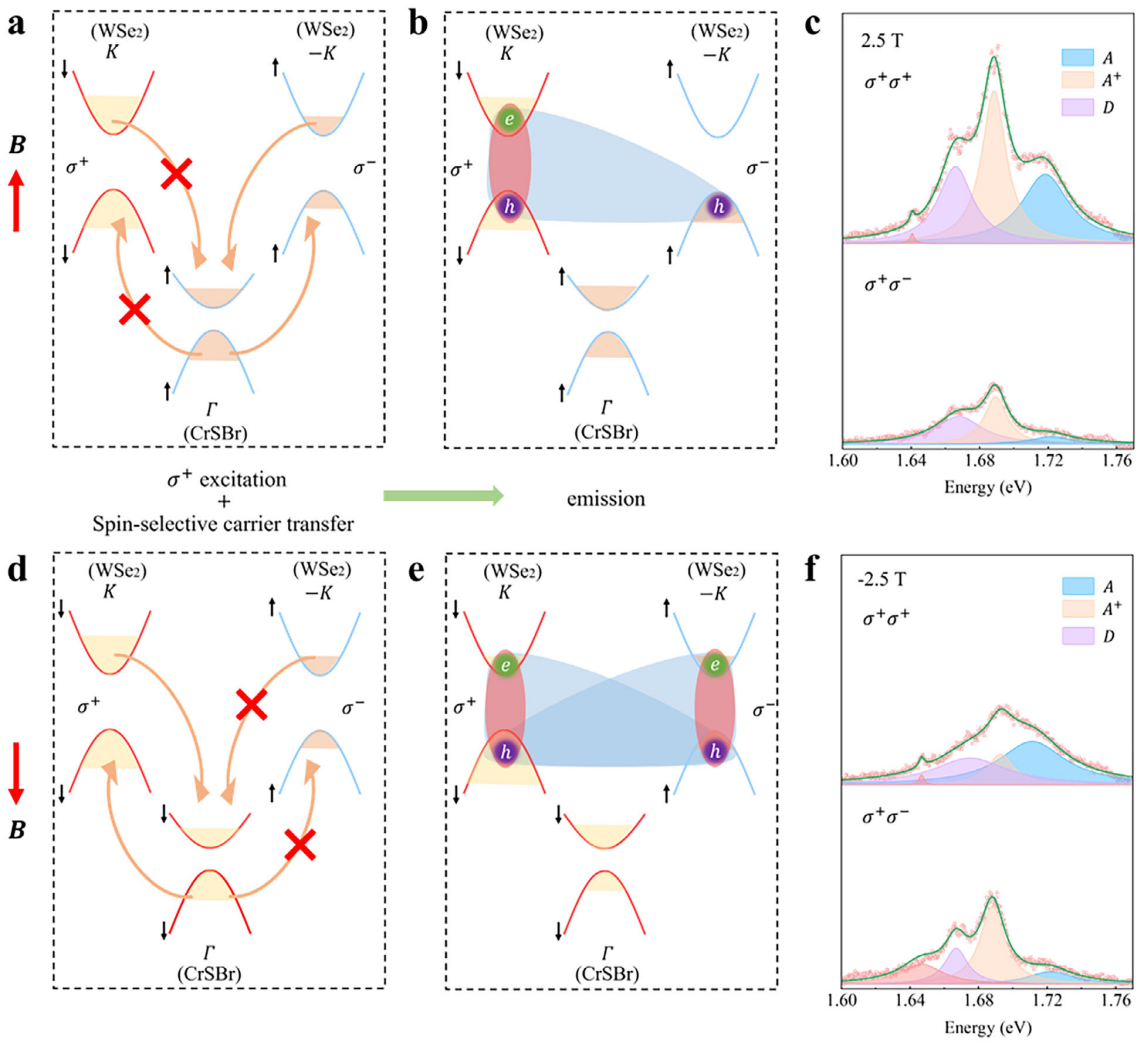
Spin‐selective hot‐carrier transfer mechanism in CrSBr/1L‐WSe_2_/CrSBr heterostructures. (a) Carrier populations immediately after σ^+^ excitation at *B_z_* > 0. Curved orange arrows indicate interfacial hot‐carrier transfer. Black arrows denote the spin orientation of the relevant bands. Red crosses mark spin‐forbidden transfer channels. (b) Carrier redistribution after spin‐selective carrier transfer, highlighting dominant recombination pathways for exciton (red oval) and trion (blue triangle) emission. (c) Representative PL spectra at *B_z_* = 2.5 T measured in σ^+^σ^+^ (top) and σ^+^σ^−^ (bottom) configurations together with Lorentzian fits of the A‐exciton (*A*; blue), trion (*A^+^*; orange), and dark exciton (*D*; purple). A weak additional feature near 1.645 eV is not discussed further. (d, e) Same as panels (a, b), but for *B_z_* < 0, where the allowed spin‐selective transfer channels are reversed. (f) Same as panel (c), but at *B_z_* = −2.5 T.

Measurements under σ^−^ excitation exhibit analogous trends, supporting the same spin‐selective hot‐carrier transfer mechanism under reversed optical initialization (Figure S20). From a symmetry viewpoint, σ^+^(σ^−^) excitation initializes an imbalance between *K* and *‐K* valleys, thereby breaking time‐reversal symmetry. Then combined with the field‐dependent spin polarization in CrSBr, this naturally leads to the observed asymmetric PL response of WSe_2_ to positive and negative *B_z_* across all four excitation/detection combinations, while keep symmetrical behavior for σ^+^σ^+^ (σ^+^σ^−^) with increasing magnetic field and σ^−^σ^−^ (σ^−^σ^+^) with decreasing magnetic field (Figures 4a–d).

### 2.5 DoCP and High Magnetic‐Field Susceptibility

We next quantify the degree of circular polarization (DoCP), defined as $DoCP=\frac{I^{\sigma^{+}}-I^{\sigma^{-}}}{I^{\sigma^{+}}+I^{\sigma^{-}}}$, where $I^{\sigma^{\pm }}$ denotes the PL intensity detected in the σ^±^ channel under a fixed excitation helicity. DoCP thus quantifies the emission helicity retention and serves as a proxy for valley polarization, with larger values indicating improved suitability for valley‐based information encoding. It should be noted that DoCP differs from helicity contrast $\rho =\frac{I^{\sigma^{+}\sigma^{+}}-I^{\sigma^{-}\sigma^{-}}}{I^{\sigma^{+}\sigma^{+}}+I^{\sigma^{-}\sigma^{-}}}$, where $I^{\sigma^{\pm }\sigma^{\pm }}$ represents the co‐helicity emission under σ^±^ excitation. ρ is often used to quantify valley imbalance arising from Zeeman splitting or magnetic proximity effects [17, 18, 19, 20]. The field‐dependent DoCP spectra are summarized in Figure 6a,b, and the integrated DoCP for *A*, *A^+^* and *D* of WSe_2_ are plotted in Figures 6c,d and S21, respectively.

**FIGURE 6:**
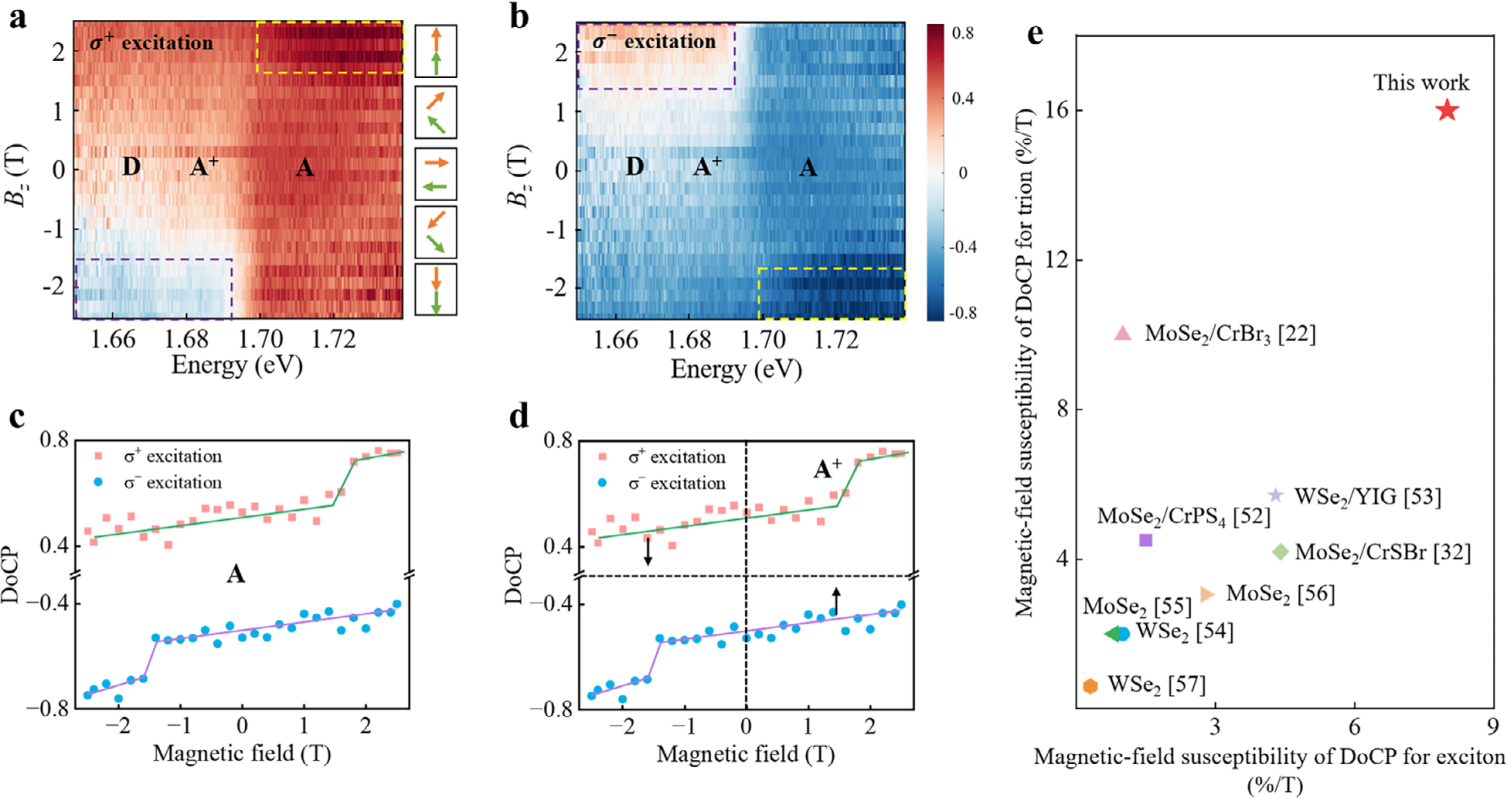
Magnetic‐field dependence of the degree of circular polarization (DoCP). (a, b) Spectrally resolved DoCP maps as a function of out‐of‐plane magnetic field *B_z_* under σ^+^ (a) and σ^−^ (b) excitation. The labels D, A^+^, and A indicate the spectral windows used for WSe_2_ dark exciton, trion, and bright A‐exciton, respectively. Panels (a, b) share the same color scale. The schematic to the right of panel (a) illustrates the corresponding field‐dependent spin configuration of CrSBr. Yellow and purple dashed rectangles mark the abrupt DoCP change and the sign reversal, respectively. (c) Spectrally integrated DoCP of the A exciton versus *B_z_* under σ^+^ (pink) and σ^−^ (blue) excitation, showing a continuous, monotonic evolution, with an abrupt change near |*B_z_* | ≈ 1.8 T. (d) Spectrally integrated DoCP of the A^+^ feature versus *B_z_* under σ^+^ (pink) and σ^−^ (blue) excitation. The DoCP varies strongly with field and reverses sign near *B_z_* ≈ ∓1.5 T for σ^±^ excitation. The distinct slopes for *B_z_* > 0 and *B_z_* < 0 are consistent with field‐sign‐dependent spin‐selective transfer pathways (Figure 4). (e) Comparison of magnetic‐field susceptibility of valley control for representative semiconductor‐magnet vdW interfaces [22, 32, 52, 53] and bare monolayer TMDs [54, 55, 56, 57]. The susceptibility is defined as ΔDoCP/Δ*B*, derived from the magnetic‐field dependence of the emission DoCP. The CrSBr/WSe_2_/CrSBr platform exhibits the highest susceptibility for both exciton and trion emission (red star).

In previously studied magnetic/TMD heterostructures, such as CrI_3_/WSe_2_ [18, 19], CrBr_3_/MoSe_2_ [21–23], Fe_3_GeTe_2_/WSe_2_ [24, 25], or Fe_3_GaTe_2_/WS_2_ [26], the magnetic layer typically possesses an out‐of‐plane easy axis, so that sweeping *B_z_* mainly switches between two spin configurations, leading to an essentially binary (spin‐up/spin‐down) modulation of the valley polarization. In contrast, in the CrSBr/WSe_2_/CrSBr heterostructure, the DoCP of both *A* and *A^+^* features evolve monotonically and continuously as *B_z_* is swept from negative to positive fields, evidencing a continuously tunable valley/spin imbalance mediated by interfacial transfer as shown in Figure 6c,d. Notably, the DoCP of *A* exhibits an abrupt change near *B_z_* ≈ ±1.8 T under σ^±^ excitation (highlighted by the yellow dashed boxes in Figure 6a,b), coincident with the field scale where the CrSBr spin configuration becomes fully aligned with *B_z_* (Figure 4). Taking σ^+^ excitation as an example, for *B_z_* > 1.8 T, the fully spin‐aligned CrSBr state maximizes spin selectivity of interlayer transfer, strongly suppressing the cross‐helicity emission and thereby producing a sharp enhancement of DoCP in *A* of WSe_2_ (Figure 6a,c). For the *A^+^* and *D* features, the DoCP not only varies strongly with field, but also reverses sign near *B_z_* ≈ ∓1.5 T and *B_z_* ≈ ∓1T, respectively for σ^±^ excitation (purple dashed rectangles in Figure 6a,b), arrows in Figures 6d and S21. These sign reversals indicate that the spin‐selective hot‐carrier transfer reshapes the relative populations contributing to the *A^+^* and *D* emission channels and can even invert the dominant helicity of the emitted PL. We also observe different DoCP slopes of *A^+^* for positive and negative fields (e.g., under σ^+^ excitation), consistent with the field‐sign‐dependent transfer pathways discussed in Figure 5.

To evaluate the possible contribution of valley Zeeman splitting, we further extracted the σ^+^/σ^−^ energy splitting of the A^+^ feature (Figure S22). The extracted effective g‐factors, *g* = −10.5 ± 2.3 and − 8.7 ± 1.8 for σ^+^ and σ^−^ excitation, respectively, indicate an approximately twofold enhancement compared with bare monolayer WSe_2_ (*g* ≈ −5) (Figure S14). Nevertheless, the measured splitting evolves approximately linearly with magnetic field, whereas the DoCP exhibits a much stronger response, including the characteristic change near ∣*B*∣ ≈ 1.8 *T* and pronounced excitation‐helicity dependence. The proximity‐enhanced valley Zeeman effect, therefore provides only a secondary contribution to the observed DoCP modulation. We quantify the magnetic‐field susceptibility of valley control as the change in DoCP per unit magnetic field, ΔDoCP/Δ*B*. This operational metric is derived from the magnetic‐field dependence of the emission DoCP, which serves as a proxy for the valley polarization rather than a direct measurement of the valley polarization itself.

The efficient spin‐selective interfacial transfer enables a giant and highly susceptible valley polarization control in WSe_2_. Across *B_z_* = ±2.5 T, the DoCP of *A* is tuned by ∼ 40%, about an order of magnitude larger than in bare monolayer WSe_2_ under comparable fields (Figure S14). For the *A^+^*, the DoCP reaches up to ∼ 80% tunability and can even change sign, corresponding to an enhancement by roughly one to two orders of magnitude compared with bare WSe_2_ or previous semiconductor–magnet van der Waals heterostructures. Figure 6e summarizes the magnetic‐field susceptibility of valley control (ΔDoCP/Δ*B*) across representative semiconductor‐magnetic vdW interfaces, highlighting the efficient and continuous tunability enabled by the CrSBr/WSe_2_/CrSBr platform.

More generally, the spin‐selective interfacial‐transfer mechanism is not restricted to the present sandwich geometry, but should be applicable to other TMD/CrSBr heterostructures with suitable band alignment and spin‐valley coupling. The magnitude and field range of the valley response are expected to depend on the TMD electronic structure and the CrSBr thickness, which determines the magnetic configuration and characteristic spin‐alignment field.

## 3 Conclusions

In summary, we have successfully demonstrated that interfacial hot‐carrier transfer provides an efficient handle to reshape the valley degrees of freedom in monolayer TMDs. Linear polarization‐resolved PL reveals a suppressed degree of linear polarization in CrSBr/WSe_2_/CrSBr heterostructures, indicating that charge transfer across the interface introduces an additional valley‐depolarization and dephasing channel that breaks valley coherence. More importantly, valley‐resolved magneto‐PL uncovers a pronounced field‐sign‐asymmetric spectral evolution that tracks the magnetic configuration of multilayer CrSBr, identifying spin‐polarized interlayer hot‐carrier transfer as the dominant underlying mechanism. This spin‐selective transfer enables continuous, high‐susceptibility control of valley polarization and coherence, including magnetic‐state‐dependent reversal and large tunability of DoCP. Our findings highlight antiferromagnet‐based vdW stacks as a robust and versatile platform for magnetically programmable quantum optoelectronic functionalities.

## 4 Methods

### 4.1 Device Fabrication

Bulk CrSBr and WSe_2_ single crystals were purchased from HQ Graphene. Thin flakes were mechanically exfoliated from the bulk crystals using Scotch tape and deposited onto Si/SiO_2_ substrates. VdW heterostructures were assembled in a nitrogen‐filled glovebox (O_2_< 3 ppm and H_2_O < 1 ppm) using a polymer‐based dry‐transfer method. A poly(bisphenol A carbonate) (PC)/polydimethylsiloxane (PDMS) stamp was used to sequentially pick up the top CrSBr flake and a monolayer WSe_2_ flake, which were then released onto a bottom CrSBr flake to form the CrSBr/WSe_2_/CrSBr stack. After transfer, the PC layer was removed by immersing the sample in chloroform for 5 min inside the glovebox. The assembled heterostructure was subsequently annealed at 250 °C for 2 h under a N_2_/H_2_ atmosphere to improve interlayer contact and reduce residual contamination. The top and bottom CrSBr flakes were aligned to the same crystallographic orientation by matching their straight cleaved edges (rectangular flake geometry). The crystallographic orientation was independently verified by excitation‐polarization‐dependent Raman spectroscopy, while the highly anisotropic CrSBr PL and cross‐sectional HAADF‐STEM measurements further support a nearly parallel crystallographic alignment of the two CrSBr flakes (Figure S2).

### 4.2 TEM Measurements

Cross‐sectional TEM lamellae were prepared using a Tescan‐GAIA focused ion beam (FIB) system equipped with a Ga ion gun. An initial protective C/Pt layer was deposited by electron‐beam deposition for ~5 min, followed by ion‐beam‐assisted Pt deposition at 30 kV using beam currents of 50‐150 pA. Initial trenching was performed at 30 kV and 2.5 nA. The lamellae were subsequently thinned at 30 kV using progressively lower beam currents of 350, 150, 50, and 20 pA. Final low‐kV polishing was performed at 5 kV/8 pA and 2 kV/50 pA to reduce FIB‐induced surface damage.

HAADF‐STEM imaging was performed using a JEOL JEM‐ARM300F2 microscope operated at an accelerating voltage of 300 kV and equipped with a cold field‐emission gun (cold FEG) and probe and image aberration correctors for STEM and TEM. The HAADF‐STEM images were acquired using an electron beam with a convergence semi‐angle of ~24 mrad and a probe current of ~23 pA. An ADF (annular dark field) detector with a camera length of 8 cm was used, corresponding to collection semi‐angles of ~79–104 mrad.

### 4.3 Low‐Temperature Magneto‐Optical Spectroscopy

Raman and PL measurements were performed in a home‐built confocal microscope in a reflection geometry, with the sample mounted in a closed‐cycle cryostat (Quantum Design OptiCool) with controlled temperature and an out‐of‐plane magnetic field. Excitation was provided by a He‐Ne tube laser (632.8 nm) focused to a spot radius of ∼ 2 µm on the sample. The backscattered signal was collected by the same objective (Mitutoyo Apochromatic NIR Objectives, 50  ×, 0.42 NA), filtered spatially using two objectives and a pinhole placed before the spectrometer to enhance the signal‐to‐noise ratio and analyzed by a grating spectrometer (Andor Kymera‐328i) equipped with a silicon EMCCD camera (Andor Newton).

For crystallographic‐axis determination, excitation‐polarization‐dependent Raman measurements were performed under 632.8 nm excitation by rotating the incident linear polarization, while collecting the Raman signal without polarization selection.

For linear polarization‐resolved PL measurements, the excitation power was kept at ∼100 µW. A half‐wave plate (HWP) was placed in the common beam path before the objective to rotate the excitation polarization while simultaneously rotating the detection basis. A Wollaston prism placed before the spectrometer separated the emitted light into two orthogonal linear polarization components, enabling simultaneous acquisition of the co‐polarized (XX) and cross‐polarized (XY) spectra (Figure S1a).

For circular polarization‐resolved PL measurements, the excitation power was kept at ∼10 µW, circularly polarized excitation was generated using a linear polarizer followed by a quarter‐wave plate (QWP). In detection, a QWP together with a Wollaston prism were used to separate the left‐ (σ^+^) and right‐ (σ^−^) circularly polarized emission components and record them simultaneously (Figure S1b).

### 4.4 Ultrafast Optical Measurements

Pump‐probe: Femtosecond pulses at 852 nm (∼120 fs pulse duration) are generated by a mode‐locked oscillator (Coherent Mira900, 76 MHz repetition rate), splitted by a beam splitter into two beams. One beam serves directly as pump beam for low‐energy pump experiments. For higher pump energies, the beam is directed through a BBO crystal for frequency doubling to 426 nm (2.91 eV). The other beam is focused into a nonlinear optical fiber (NKT PM‐750) to yield super continuum white light(470–850 nm) as probe. The pump‐probe time delay is controlled by a motorized stage. Both beams are focused onto the sample to a spot size of ∼ 2–3 µm. The reflected probe beam is collected and directed to a spectrometer (Andor shamrock193) equipped with a CCD (Newton EMCCD) for spectral acquisition. A mechanical shutter is used to alternately block and unblock the pump beam. The reflection intensity difference (dR) is extracted by reducing the probe spectra acquired with and without pump excitation.

### 4.5 Time‐Correlated Single Photon Counting (TCSPC)

Time‐resolved photoluminescence (TRPL) measurements were performed using a SPC‐130‐EMN Module (Becker & Hickl GmbH). Femtosecond pulses at 488 nm and 800 µW serves as the excitation. The 76 MHz synchronization signal from the femtosecond oscillator is directly input to the sync port of the TCSPC module. Photoluminescence photons are detected by a single‐photon avalanche detector (MPD series, ∼50 ps time resolution), converted to NIM signals that enter the CFD port. The time difference between each excitation pulse and the detected PL photon is recorded to construct a histogram for yielding the time‐resolved PL decay trace.

## Author Contributions

K.X. and S.S.P.P. conceived of the project. K.X. set up the low‐temperature magneto‐optical spectroscopy with the help of Y.G. K.X. performed the PL measurements, analyzed the data and set up the theoretical model. J.Y. prepared the sample and fabricated the heterostructures. K.F. performed ultrafast optical measurement. R.H. prepared the sample for TEM measurements and J.P. performed the TEM measurements. K.X. and S.S.P.P. wrote the manuscript with input from all the authors. All the authors discussed the results.

## Conflicts of Interest

The authors declare no conflicts of interest.

## Supporting information




**Supporting File**: adma75103‐sup‐0001‐SuppMat.docx.

## Data Availability

The data that support the findings of this study are available from the corresponding author upon reasonable request.
