# Supplementary material for: Giant Nonequilibrium Valley Control via Spin‐Selective Hot‐Carrier Transfer in an Antiferromagnetic van der Waals Stack
Source: Adv Mater. 2026 Sep 29;38(63):e75103. doi: 10.1002/adma.75103 (PMC13755652; doi:10.1002/adma.75103)
Supplement: Supplementary file 1 [file ADMA-38-e75103-s001.docx]

**Giant nonequilibrium valley control via spin-selective hot-carrier transfer in an antiferromagnetic van der Waals stack**

Ke Xiao^1 *^, Jiabao Yang^1^, Kai Feng^2^, Yicheng Guan^1^, James Caleb Peters^1^, Ramona Hoffmann^1^, Niels Schröter^1,3^, Stuart S. P. Parkin^1*^

^1^ NISE department, Max Planck Institute of Microstructure Physics, Halle (Saale) 06120, Germany

^2^ Physics Department and state key laboratory of quantum optical materials , The University of Hong Kong, Pokfulam road, Hong Kong, China

^3^ Martin-Luther-Universität Halle-Wittenberg, Halle (Saale), Germany

Corresponding authors: [ke.xiao@mpi-halle.mpg.de](mailto:ke.xiao@mpi-halle.mpg.de); [stuart.parkin@mpi-halle.mpg.de](mailto:stuart.parkin@mpi-halle.mpg.de)

**Supplementary Figure 1: Optical Setup for low-temperature magneto-optical spectroscopy** (a) Schematic of the set-up for linear polarization-resolved PL measurements. (b) Schematic of the set-up for circular polarization-resolved PL measurements. Abbreviations: EMCCD: electron-multiplying charge-coupled device; WP: Wollaston prism; O: objective; PH: pinhole; NBS: non-polarizing beam splitter; LP: linear polarizer; HWP: half-wave plate; QWP: quarter-wave plate.

**Supplementary Figure 2. Structural characterization of the CrSBr/WSe_2_/CrSBr device.** (a) Optical image with bottom CrSBr (yellow), top CrSBr (orange) and monolayer WSe_2_ (red) outlined; the white line marks the AFM line-cut. (b) AFM topography. (c) Height profile along the line-cut: 8.1 nm (bottom CrSBr, ~10 layers), 10.6 nm (top CrSBr, ~13 layers) and 1.5 nm (monolayer WSe_2_ plus van der Waals gaps). (d) Low-magnification cross-sectional HAADF-STEM image of the stack. (e) Atomic-resolution image of the CrSBr/WSe_2_/CrSBr interface region; red lines mark the van der Waals gaps. (f) Atomic-resolution HAADF-STEM image of CrSBr with the atomic model overlaid (Cr: blue, S: yellow, Br: orange).

**Supplementary Figure 3.** **Determination of the relative crystallographic orientation of the CrSBr flakes**. Polar plots of the integrated $A_{g}^{2}$ Raman intensity measured from the **top CrSBr (a) and bottom CrSBr** (b) under 633 nm excitation. Only the linear polarization of the excitation was rotated, while the Raman signal was collected without polarization selection. The red dots represent the excitation spots. For both flakes, the $A_{g}^{2}$ mode reaches its maximum along the crystallographic b-axis. The nearly identical angular positions of the maxima demonstrate the nearly parallel alignment of the two CrSBr flakes.

**Supplementary Figure 4: In-plane PL anisotropy of the CrSBr/WSe_2_/CrSBr heterostructure** (a) Polar plot of the integrated PL intensity of the CrSBr emission peak measured in the co-polarized (XX) and cross-polarized (XY) configurations (b) Polar plot of the integrated PL intensity of WSe_2_ emission peak in the XX and XY configurations. Dashed lines indicate the crystallographic *a* and *b* axes of CrSBr.

**Supplementary Figure 5: Valley-resolved type-III (broken-gap) band alignment of the CrSBr/WSe_2_/CrSBr heterostructure.** The left and right columns show the K and −K valleys of monolayer WSe_2_ (red and blue); the central column shows CrSBr (gold). Energies are referenced to the vacuum level; the CrSBr conduction-band minimum lies ~0.18 eV below the WSe_2_ valence-band maximum.

**Table S1: Band-edge energies of WSe_2_ and CrSBr referenced to the vacuum level**.

**Supplementary Figure 6: Suppression of valley coherence by hot carriers transfer.** (a) Excitation-power-dependent PL spectra measured in the co-polarized (XX) configuration. (b) Excitation-power-dependent PL spectra measured in the cross-polarized (XY) configuration. Panels (a) and (b) share the same color scale shown to the right of panel (b). (c) DoLP spectra of WSe_2_ as a function of excitation power. The trion (*A^+^*) feature exhibits a consistently lower DoLP, while the *A* DoLP decreases with increasing excitation power, consistent with enhanced interfacial hot-carrier transfer and valley dephasing. (d) DoLP spectra of CrSBr as a function of excitation power, remaining nearly unchanged at a high level across the measured power range.

**Supplementary Figure 7: Characteristic timescales of the relevant photoexcited-carrier and exciton processes.**

**Supplementary Figure 8. Interfacial carrier extraction under simultaneous excitation of WSe_2_ and CrSBr.** (a) Energy-level scheme. A 2.91 eV (426 nm) pump lies well above the optical gaps of both WSe_2_ (~1.72 eV) and CrSBr (~1.36 eV), thereby photoexciting both materials. Under the type-III (broken-gap) alignment, electrons transfer from the WSe_2_ conduction band to CrSBr, while holes transfer from CrSBr into the WSe_2_ valence band (blue arrows). The dynamics are probed through the WSe_2_ A-exciton response at ~1.72 eV. (b) Transient A-exciton signal, |dR|/R of bare WSe_2_ (green) and the CrSBr/WSe_2_/CrSBr heterostructure (red), measured under identical conditions. The A-exciton signal in the heterostructure is reduced to approximately one third of that in bare WSe_2_, consistent with an additional ultrafast interfacial carrier-transfer channel that competes with the formation and population of WSe_2_ excitons.

**Supplementary Figure 9: Time-resolved photoluminescence measurements**. (a) TCSPC trace of the normalized WSe_2_ A-exciton emission at 725nm. The grey solid line represents the instrument response function and the green line shows a biexponential fit. (b) Enlarged view of the early-time region marked by the dashed box in (a). The emission exhibits an instrument-limited fast decay component of $\tau_{1}<20$ ps and a slower decay component of $\tau_{2}=303\pm16$ ps which correspond to exciton radiative recombination and exciton lifetime, respectively, inconsistent with the characteristic timescales summarized in **Supplementary Figure 7**.

**Supplementary Figure 10: Circular polarization analysis is insensitive to Faraday rotation of the cryostat windows.** (a-c) 633-nm laser-line profiles (Rayleigh signal) recorded under $\sigma^{+}$ excitation with detection in $\sigma^{+}$ (black) and $\sigma^{-}$ (red) channels at -3 T (a), 0 T (b) and 3 T (c), respectively. The $\sigma^{+}\sigma^{+}$ signal dominants with negligible leakage into $\sigma^{+}\sigma^{-}$ channel under these representative fields. (d-f) Same measurement under $\sigma^{-}$ excitation with detection in $\sigma^{+}$ (black) and $\sigma^{-}$ (red) channels at -3 T (d), 0 T (e) and 3 T (f), respectively. The $\sigma^{-}\sigma^{-}$ signal dominants with negligible leakage into $\sigma^{-}\sigma^{+}$ channel under these representative fields, confirming that the measured circular polarization is not affected by Faraday rotation of the optical windows.

**Supplementary Figure 11: Valley-resolved PL spectra in CrSBr/1L-WSe2/CrSBr at zero magnetic field.** (a-d) PL spectra detected in the $\sigma^{-}$ (black) and $\sigma^{+}$ (red) channels together with the corresponding spectrally resolved DoCP (blue symbols), measured at $B_{z}=0$ under $\sigma^{-}$ excitation (a), $\sigma^{+}$ excitation (b), a-axis linear excitation (c) and b-axis linear excitation (d). Three characteristic features are resolved and assigned to bright A-exciton (*A*, green), trion (*A^+^*, dark red) and dark exciton (*D*, pink). The trion-to-exciton intensity ratio is higher for b-axis excitation than for a-axis excitation, consistent with stronger interfacial hole transfer from CrSBr to WSe_2_ under b-axis excitation, where CrSBr absorption is enhanced. Panels (a) and (b) are nearly symmetric, indicating that the hot-carrier transfer is helicity independent at zero magnetic field.

**Supplementary Figure 12: Valley-resolved PL spectra in *h*BN/1L-WSe_2_/*h*BN at zero magnetic field.** (a-c) PL spectra detected in the $\sigma^{-}$ (black) and $\sigma^{+}$ (red) channels together with the corresponding spectrally resolved DoCP (blue symbols), measured at $B_{z}=0$ under $\sigma^{-}$ excitation (a), linearly polarized excitation (b) and $\sigma^{+}$ excitation (c). Two characteristic features are resolved and assigned to bright A-exciton (*A*, green), trion (*AT*, pink). No dark-exciton-related feature (*D*) is observed in the *h*BN/1L-WSe_2_/*h*BN control sample, consistent with the *D* peak emerging in the CrSBr/1L-WSe_2_/CrSBr heterostructure due to proximity effects at the antiferromagnet–semiconductor interface.

**Supplementary Figure 13: Circular-polarization-resolved magneto-photoluminescence of bare monolayer WSe_2_ at 2 K.** Magnetic-field-dependent PL intensity maps measured under the four excitation/detection helicity combinations: (a) $\sigma^{+}\sigma^{+}$, (b) $\sigma^{+}\sigma^{-}$, (c) $\sigma^{-}\sigma^{+}$, (d) $\sigma^{-}\sigma^{-}$, where first and second symbols denote the excitation and detection helicity, respectively). The out-of-plane magnetic field $B_{z}$ is swept from −3 to +3 T.

**Supplementary Figure 14: Magnetic-field dependence of the A exciton in bare monolayer WSe_2_ at 2 K.** (a) Representative PL spectrum, resolving the A exciton (~1.742 eV) and a broader lower-energy band assigned to trion and defect-related emission. (b) Spectrally integrated PL intensity of the A exciton for the four helicity combinations ($\sigma^{+}\sigma^{+}$, $\sigma^{-}\sigma^{-}$, $\sigma^{+}\sigma^{-}$, $\sigma^{-}\sigma^{+}$) versus magnetic field. (c) DoCP of the A exciton as a function of out-of-plane magnetic field under $\sigma^{+}$ (pink) and $\sigma^{-}$ (blue) excitation. (d) Valley Zeeman splitting, $\Delta E = E(\sigma⁺) - E(\sigma⁻)$ of the A exciton versus magnetic field; the solid lines are linear fits yielding an A-exciton g-factor of $g\approx4.9$or $5.3$.

**Supplementary Figure 15: Circular-polarization-resolved magneto-PL of the CrSBr/WSe_2_/CrSBr heterostructure at 50 K.** Magnetic-field-dependent PL intensity maps measured under the four excitation/detection helicity combinations: (a) $\sigma^{+}\sigma^{+}$, (b) $\sigma^{+}\sigma^{-}$, (c) $\sigma^{-}\sigma^{+}$, (d) $\sigma^{-}\sigma^{-}$, where the first and second symbols denote the excitation and detection helicity, respectively. The out-of-plane magnetic field $B_{z}$ is swept from −3 to +3 T. The red line is a guide to the eye tracking the field-dependent WSe2 emission energy.

**Supplementary Figure 16: Temperature dependence of the PL response of the CrSBr/WSe_2_/CrSBr heterostructure.** (a) Normalized PL spectra at 2 K and 50 K in the $\sigma^{+}\sigma^{+}$ and $\sigma^{+}\sigma^{-}$ configurations, showing the thermal merging of the trion and A-exciton into a single feature at 50 K. (b, c) Spectrally integrated PL intensity of the four helicity combinations ($\sigma^{+}\sigma^{+}$, $\sigma^{+}\sigma^{-}$, $\sigma^{-}\sigma^{+}$, $\sigma^{-}\sigma^{-}$) as a function of out-of-plane magnetic field at (b) 50 K and (c) 2 K.

**Supplementary Figure 17. Spatial reproducibility of the circular-polarization-resolved PL.** (a) Optical image of the heterostructure with the five measured positions (scale bar 10 µm). (b, c) Normalized zero-field PL spectra at P1-P5 under (b) $\sigma⁺$and (c) $\sigma⁻$ excitation; red and blue denote $\sigma⁺$ and $\sigma⁻$ detection. (d) Integrated PL intensity in the four helicity channels at P1-P5. (e) Zero-field DoCP at P1-P5 under $\sigma⁺$ and $\sigma⁻$ excitation. (f) DoCP versus out-of-plane magnetic field at P1-P3 under $\sigma⁺$ (red/orange) and $\sigma⁻$ (blue/purple) excitation.

**Supplementary Figure 19: Spatial reproducibility of the linear-polarization anisotropy.** (a) Polarization-angle-resolved DoLP of CrSBr (blue) and WSe_2_ (red) at position P3. (b) DoLP values along the a- and b-axes at PM (main text) and P3.

**Supplementary Figure 20: Spin-selective hot-carrier transfer mechanism in CrSBr/1L-WSe_2_/CrSBr heterostructure.** (a) Carrier populations immediately after $\sigma^{-}$ excitation at $B_{z}>0$. Curved orange arrows indicate interfacial hot-carrier transfer. Black arrows denote the spin orientation of the relevant bands. Red crosses mark spin-forbidden transfer channels. (b) Carrier redistribution after spin-selective carrier transfer, highlighting dominant recombination pathways for exciton (red oval) and trion (blue triangle) emission. (c) Representative PL spectra at $B_{z}=2.5$ T measured in $\sigma^{-}\sigma^{+}$ (top) and $\sigma^{-}\sigma^{-}$ (bottom) configurations, together with Lorentzian fits of the A exciton (*A*; blue), trion (*A^+^*; orange) and dark exciton (*D*; purple). A weak additional feature near 1.645 eV is not discussed further. (d-e) Same as panels (a, b), but for $B_{z}<0$, where the allowed spin-selective transfer channels are reversed. (f) same as panel (c), but at $B_{z}=-2.5$ T.

**Supplementary Figure 21: Spectrally integrated DoCP of the dark-exciton-related feature.** Spectrally integrated DoCP of the D feature versus $B_{z}$ under $\sigma^{+}$ (pink) and $\sigma^{-}$ (blue) excitation. The DoCP varies strongly with field and reverses sign near $B_{z}\approx\mp1$T for $\sigma^{\pm}$ excitation, consistent with spin-selective hot-carrier transfer that redistributes valley/spin populations and can invert the dominant emission helicity of the D channel.

**Supplementary Figure 22. Magnetic-field-dependent valley Zeeman splitting in the CrSBr/WSe_2_/CrSBr heterostructure.** Energy splitting $\Delta E=E(\sigma^{+})-E(\sigma^{-})$ of the A⁺ feature as a function of magnetic field under $\sigma^{+}$ and $\sigma^{-}$ excitation. Solid lines represent linear fits used to extract the effective g-factors. The corresponding value for bare monolayer WSe_2_ ($g\approx-5$) is shown for comparison.
